# Understanding the bricks to build better surgical oncology unit at Maputo Central Hospital: prevalent surgical cancers and residents knowledge

**DOI:** 10.11604/pamj.2019.32.83.18126

**Published:** 2019-02-18

**Authors:** Atílio Morais, Jotamo Come, Carlos Selemane, Germano Pires, Adriano Tivane, Matchecane Cossa, Satish Tulsidás, Luís Antunes, Manuel João Costa, Moshin Sidat, Maria do Rosário Martins, Carla Carrilho, Lúcio Lara Santos

**Affiliations:** 1 Surgical Department, Maputo Central Hospital, Maputo, Mozambique; 2 Department of Surgery of Faculty of Medicine, University of Eduardo Mondlane, Maputo, Mozambique; 3 Medical Oncology Department, Maputo Central Hospital, Maputo, Mozambique; 4 Epidemiology Service, Portuguese Institute of Oncology, Porto, Portugal; 5 Medical Education Unit, School of Medicine University of Minho, Portugal; 6 Department of Community Health, University of Eduardo Mondlane, Maputo, Mozambique; 7 Global Health and Tropical Medicine, Institute of Hygiene and Tropical Medicine, NOVA University of Lisbon, Portugal; 8 Pathology Department, Maputo Central Hospital, Maputo, Mozambique; 9 Surgical Oncology Department, Portuguese Institute of Oncology, Porto, Portugal; 10 Experimental Pathology and Therapeutics Research Group, Portuguese Institute of Oncology, Porto, Portugal; 11 ONCOCIR- Education and Care in Oncology, Lusophone, Africa

**Keywords:** Cancer surgery, Resident knowledge, Mozambique

## Abstract

**Introduction:**

Cancer is a growing concern in Mozambique. However, the country has limited facilities and few oncologists. Surgical oncologists are an unmet need. The aim of this study was to assess residents' knowledge in prevalent cancer domains and to identify and characterize prevalent cancers treated by surgery at Maputo Central Hospital, the largest hospital in Mozambique. The expectations were that the findings shall inform the development of a comprehensive curriculum in surgical oncology fellowship fit for the Hospital.

**Methods:**

To identify and characterize prevalent cancers, we performed a retrospective analysis of individual cancer patient registries of Maputo Central Hospital (MCH), Mozambique. Information was recorded into data collection sheets and analyzed with SPSS^®^ 21. To assess MCH residents oncologic knowledge, we invited Twenty-six junior residents (49% of all residents) of different specialties to take a 30 item multiple choice written test used elsewhere in previous studies. The test focused on the domains of Basis of oncology, Radiotherapy, Pathology, Chemotherapy, Pain management, Surgical oncology and Clinical Pathway. The test was administered anonymously and without prior notice. We analyzed the overall test and topic performance of residents.

**Results:**

The study covered a period of 3 years and 203 patients. The most prevalent malignant tumors treated by general and thoracic surgery in MCH cancer registry were esophageal (7%), female breast (6.5%) and colorectal cancer (2.8%). Globally these malignancies were diagnosed at an advanced stage of the disease and required a multimodal treatment. The mean percent correct score of residents was 37.3%. The dimension with the highest percent correct score were clinical management (46%) and surgical oncology (28%) showed the lowest correct score.

**Conclusion:**

In Maputo, Mozambique esophageal, breast and colorectal cancer were the most prevalent malignancies treated, with surgery, by thoracic or general surgery in MCH. The test scores suggest that, among residents, the knowledge in oncology needs to be improved, rendering support to the need of a surgical oncology training tailored to suit the local needs. Specific training should take into account local cancer prevalence, resources, their quality and the support of surgical oncology services with volume and experience.

## Introduction

Cancer is a growing concern in Mozambique [1]. However, the provision of care to cancer patients is conditioned by the country limitations on facilities and specialized oncologists [2]. Patients with advanced stages poses huge challenges in terms of adequate and effective treatment [3, 4]. Cancer surgery, the oldest therapy modality remains the mainstay of treatment for solid tumors in countries with limited resources. In some less-developed regions of the world, surgery may be the only viable cancer treatment option [5]. Surgery is effective in the treatment of localized or locally advanced primary tumors and may prolong survival after surgical resection of distant metastases [6]. Thus, surgical oncology, as a subspecialty of general surgery, has emerged to play an increasingly important role in the multidisciplinary treatment of cancer. Effective cancer surgery is a combination of complex variables including the biology of the disease, patient health circumstances, and resources available, all intertwined with the surgeon's judgment and skill [7]. Unfortunately, more than three-quarters of cancer patients in low-income and middle-income countries (LMICs) do not receive timely, safe, and affordable cancer surgery [5, 8]. To address this impending problem of lack of adequate surgical care for cancer patients on the global stage, the European Society for Surgical Oncology (ESSO) and the Society of Surgical Oncology (SSO) outlined a framework of a global curriculum in surgical oncology education and training [9]. Such a framework is expected to enhance the quality of the specialists. However, the number of cancer surgeons that successfully accomplished this curriculum is insufficient. Therefore, this is an unmet need [10]. According to Murray Brennan of Memorial Sloan-Kettering Cancer Center in New York, cancer surgeon need to: (1) understand etiology and genetic predisposition; (2) understand prognostic factors and natural history; (3) perform cost-effective treatment; (4) develop clinical trials; (5) guides advanced disease management; (6) guide compassionate support; and (7) evaluates outcome [11]. Sullivan R *et al*. argued that there are no one-size-fits-all system solutions to train surgical oncologist, but because surgery is so central to patient outcomes, focusing on this as a central part of national cancer control plans is crucial [7]. In Mozambique, cancers are prevalent. For example, 2016 and 2017 statistical data from the Surgical Department of Maputo Central Hospital (MCH) show that, out of 7051 operations performed (minor procedures were excluded), 606 (8.6%) were malignancies. The most prevalent malignant tumors treated by general and thoracic surgery, according to 2015-16 MCH cancer registry (1705 patients), were esophageal (7%), female breast (6.5%) and colorectal cancer (2.8%) [3]. Recently, Morais A *et al*. studied in depth the surgical resources available at the MCH to perform oncologic surgery, the difficulties manifested by the surgeons during the surgical treatment of cancer and also designed a draft training program in cancer surgery [12]. In Mozambique, surgeons are proficient in general surgery, namely in the treatment of trauma and of surgical complications of infectious diseases. However, in what regards malignant neoplasm surgery, their knowledge and experience need to be enhanced. Therefore, is essential to train and certify surgical oncologist according to good oncological practices [13]. So, we must build our solution, taking into account the resources that we have, our cultural identity, the local nosology, the knowledge that future specialists have at the moment, the supports and partnerships that we already have and those we can involve, in order to quickly form the trainers who will ensure the future in this field. In Maputo, the training of the residents is carried out at Maputo's Central Hospital (MCH), the largest hospital in Mozambique. The development of a fit for purpose, comprehensive and fast-track training program in surgical oncology at MCH, requires the characterization of the clinical and pathological profile of the most prevalent cancer surgery at Central Hospital de Maputo. In response to the shortage of information do develop an oncology training program, this study had two primary aims, namely: I. the characterization of the most prevalent malignant tumors treated at MCH; II. surveying resident's knowledge of surgical oncology.

## Methods

**Identification of the most prevalent malignant tumors treated at the department of surgery:** we conducted a retrospective analysis using database onto cancer surgery from Maputo Central Hospital and according to a checklist for retrospective database studies [14]. Information was collected from the operating room logbooks and records of patients dated from January 1, 2014, to December 31, 2016. Cancer cases were categorized according to demographic data, organ, diagnosis, histologic type, stage and treatment (surgical and/or systemic). Histologic type and stage were classified according to WHO tumor classification and European Network of Cancer Registries (ENCR) recommendations for condensed TNM for coding the extent of disease, respectively [15-18]. Only full records were included. The three most frequent malignant tumors treated by thoracic or general surgery were also classified according to the reported intention of treatment as curative or palliative.

**Assessment of basic and clinical oncology knowledge:** twenty-six (49% of all MCH residents) residents of different specialties-general surgery, thoracic surgery, otolaryngology, medical oncology, pathology and gastroenterology- who would participate in an oncology course organized by the Surgical Department of MCH, were invited by the course promoters, to individually perform a written test. All participants were ensured that the results would not have consequences for their professional life. The test was administered under proctoring conditions with time limit of 60 minutes. These residents answered a written test, developed by LLS which had been used for similar purpose in Portugal (Master Degree in Oncology, Specialization in Clinical Oncology) and in other Portuguese speaking countries [19]. The test was composed of selected response items and assessed seven dimensions of oncology during a single test session. The test blueprint included: Basis of oncology, Radiotherapy, Pathology, Chemotherapy, Pain Management, and Clinical Pathway. The item contexts were breast, esophagus, oral cancer and colorectal cancer. The test was made of 30 items, there were 12 single best answers multiple choice, 15 multiple true/false, 2 correspondence and 1 short answer item. We calculated individual participant scores, item percent correct scores and discrimination, we also test reliability using Cronbach´s alpha. For data analysis we used descriptive statistics. The study was approved by the Mozambican National Bioethical Committee.

## Results

**Malignant tumors more prevalent treated at department of surgery:** the most common cancers treated by surgery (general or thoracic), in the study period, were in order of frequency 156 (77%) breast, 25 (12%) esophagus and 22 (11%) colorectal (Table 1). More than a half of the 25 HCM patients with esophageal cancer were women, and the median age at diagnosis of 50 years (Min 27; max 74); colorectal cancer was most prevalent in men and over the 22 patients with median 47 years (Min 24; max 83). Breast cancer patients were exclusively women with median 49 years (Min 18; max 84).

**Table 1:** Demographics characteristics and overall survival of the patients treated by the surgical department of the MCH

Variables	Patients with Esophagus cancer (n=25)		Patients with Colorectal cancer (n=22)		Patients with Breast cancer (n=156)	
**Age at diagnosis** (years)	50		47		156	
**Median** (min; max)	(min - 27; max - 74)		(min - 24; max - 83)		(min - 18; max - 84)	
**Gender**	**n**	**%**	**n**	**%**	**n**	**%**
Male	11	44.0	13	59.1	0	0
Female	14	56.0	9	40.9	156	100
**Surgical treatement intent**	**n**	**%**	**n**	**%**	**n**	**%**
Curative	0	0	4	18.2	139	89.1
Paliative	25	100.0	18	81.8	17	10.9
**Systemic treatment**	**n**	**%**	**n**	**%**	**n**	**%**
No	12	48.0	18	18.2	18	11.5
Yes	13	42.0	4	81.8	138	88.5

**Esophageal cancer:** globally these malignancies were diagnosed at an advanced stage of the disease. The late diagnosis limits the offer of surgical treatment with curative intent is associated with poor survival [20]. Regarding the location of tumor, 12 were of the middle third and 12 of the lower third and only one case was of the upper third. Twenty-three cases were squamous cell carcinomas and two were adenocarcinomas. In most tumors, the attempt of curative resection was performed and if it was not possible a palliative resection or surgical bypass was performed (n=22), in order to preserve the feeding of these patients. The Ivor-Lewis operation was the most accomplished. In the cases, without conditions for thoracotomy, we performed feeding gastrostomies. The systemic treatment used cisplatin and fluorouracil (5-FU), regardless of its setting. Radiotherapy is not yet available in HCM.

**Colorectal cancer:** in these tumors, also, diagnosis occurs in an advanced stage. Regarding the location of these tumors, 3 (13.7%) were of the right colon, 3 (13.7%) were of the left colon, 2 (9.0%) were of the sigmoid and the remaining 14 (63.6%) were of the rectum. Most tumors were adenocarcinomas (86.4%), two squamous cell carcinomas were associated with advanced anal canal carcinomas (patients 43 and 83 years old) and one case revealed that it was a lymphoma involving the colon (patient 42 years). It was only possible to perform curative surgery in 4 (18.1%) since patients are referred or reach HCM with unresectable disease. Systemic treatment used 5-FU or irinotecan (in 4 cases the intention was adjuvant). The disease stage in most cases suggests poor survival.

**Breast cancer:** in MCH, multidisciplinary oncology treatment decision on breast cancer is already established and most breast malignant tumors are treated with systemic neoadjuvant treatment since they are locally advanced malignant tumors. The modified radical mastectomy was the most frequent surgical option (88.5%). When the study was performed, we had no access to radiotherapy and so, we did not perform conservative breast surgery. The exceptions were the cases that, to complete treatment radiotherapy was performed abroad. Advanced breast cancers sometimes develop complex wounds with associated pain, infection, massive discharge, malodor and bleeding, which distresses patients. Therefore, we performed Palliative surgery in these cases, allowing the patients to feel a more comfortable. Adriamycin-Cyclophosphamide (AC) followed by Paclitaxel (PC) was the most frequent options. Cisplatin is used in triple-negative or non-responder tumors and hormone therapy is prescribed when indicated.

**Resident's knowledge:** the reliability of the test was 0,728 (which indicates an acceptable internal consistency) and all but one item showed positive discrimination indexes. Table 2 summarizes the participant overall and domain results percent correct scores. The mean percent correct score for the test was 37.3%. The dimension with the highest percent correct score were clinical management (46%) and surgical oncology (28%) showed the lowest correct score. In what concerns participant performance, the average percent correct score was 40% (Maximum= 60.0%, Minimum=13.5%).

**Table 2:** Results obtained by the 26 residents for different themes of oncology knowledge

Oncology knowledge domain	Number of questions	Correct answers	Total answers	Correct answers (%)
Basis of oncology	7	75	182	41.2%
Radiotherapy	2	12	52	23.1%
Pathology	2	15	52	28.8%
Chemotherapy	6	49	156	31.4%
Pain management	3	35	78	44.8%
Surgical oncology	3	22	78	28.8%
Clinical pathway	7	83	182	45.6%
**Total**	**30**	**291**	**780**	**37.3%**

## Discussion

In this study, we found that in Maputo, Mozambique esophageal, breast and colorectal cancer were the most prevalent malignancies treated by general and thoracic surgery in MCH (3). Main cases were locally advanced and their treatment needs high expertise and associated treatments. The finding on oncological knowledge of residents reveals that Mozambique needs to develop at the medical school level, a cancer education program. In addition, the results support the argument that a comprehensive cancer program in order to train all residents must be integrated in the residency training program. It is also necessary, train surgeons (future mentors) and residents of surgery in surgical oncology, prioritizing the most prevalent tumors in the country. Currently, the Project ECHO Mozambique that is ongoing, as a result of a collaborative effort between MD Anderson, three medical institutions in Brazil, the Maputo Central Hospital (Mozambique) and the Ministry of Health in Mozambique [21]. This partnership is working to increase clinical capacity through a comprehensive training program including regular telementoring, hands-on training workshops and professional exchanges including in cancer surgery. Additionally, the “Integrated care for the cancer patient” a program of the Calouste Gulbenkian Foundation, Portugal is in-course to strengththe institutional capacity of the Central Hospital of Maputo, Mozambique, by improving cancer education (that include cancer surgery), screening, diagnosis, treatment and registration of oncological diseases [22]. However, and according to Morais A *et al*. the absence of an agglutinative program of these interventions and their combination with the national training activities are responsible for poorly structuring results [12]. About Collaborative Programs and their effective results, we endorse the editorial recently published in JGO journal by Bishal Gyawali and Gilberto Lopes in which they refer about non-formal cancer education: “It is still an open question if these investments actually lead to better outcomes … we need to create measures and performance indicators that reflect positive changes and improvements in cancer control efforts “[23]. Kenya, for instance, to solve similar problems, evaluated with their cancer research and control stakeholder program and their national program together in order to strengthen the national cancer control plan [24]. We believe that, in addition to non-formal training, the formal and specific training of cancer education is the key and should involve local universities, colleges of surgery and scientific societies. They must have potential support of international organizations such as African Colleges of Surgery, African Organization for Research and Training in Cancer (AORTIC), ESSO and SSO. In this sense, in June 2018 in Cape Verde, the III Portuguese-speaking African Countries Oncology Congress (PALOP-AORTIC meeting) concluded that the organized and formal formation of medical doctors, surgical residents and surgical specialists along with the current non-formal training may be the better solution [25]. Thus, the development of a workforce is one of the ways of contributing to the realization of the Mozambican Initiative in Surgical Oncology (MISO), a program that we intend to develop integrating all in order to quickly fill the gap in cancer surgery. The training program for residents of surgical oncology should include, in addition to training in MCH, training in surgical oncology services with volume and experience. Morais A and colleagues pointed out the need to assure the basic requirements to treat patients with esophageal, breast and colorectal cancer, and the need to implement the notion that the therapeutic decision must be multidisciplinary [12]. This study stresses the need of a training program for residents and surgeons for delivery safe and effective cancer surgery.

## Conclusion

According to this study, surgical oncology training is crucial, and we suggest it should be condensed and tailored to suit the local needs. The oncology education program should be taught initially at the university level and improved during medical specialties. This specific training should take into account local cancer nosology, resources, their quality and the support of surgical oncology services with volume and experience. It is crucial to coordinate all training efforts efficiently so that results are relevant.

### What is known about this topic

- There is a growing incidence of cancers in Mozambique and a concern that surgical oncology training may be insufficient.

### What this study adds

- The study identified the cancers most frequently treated at MCH and revealed important gaps in resident's knowledge about surgical oncology. The findings support the need of a local curriculum and provide directions for the development of a program in surgical oncology, which might be transferrable to other developing countries, taking into account local cancer nosology, resources, knowledge of oncology among residents and stakeholder support.

## Competing interests

The authors declare no competing interests.
